# Molecular evidence of parvovirus B19 in the cutaneous polyarteritis nodosa tissue from a patient with parvovirus-associated hemophagocytic syndrome: Case report

**DOI:** 10.1097/MD.0000000000022079

**Published:** 2020-09-04

**Authors:** Ji Yun Jeong, Ji Young Park, Ji Yeon Ham, Ki Tae Kwon, Seungwoo Han

**Affiliations:** a Department of Pathology; b Departments of Clinical Pathology; c Departments of Internal Medicine, Kyungpook National University, School of Medicine, Daegu, Republic of Korea.

**Keywords:** arteritis, hemophagocytic lymphohistiocytosis, parvovirus B19, pure red cell aplasia

## Abstract

**Rationale:**

Parvovirus B19 has been linked to polyarteritis nodosa (PAN), but there is some controversy about its pathogenesis regarding whether it is triggered by the immune complex or by the activated immune cells that phagocytose viruses.

**Patient concerns:**

A 38-year-old woman was admitted with fever and bicytopenia. She also complained of a painful palpable nodule in the left forearm.

**Diagnosis:**

Her bone marrow aspirate revealed erythroblasts in abnormal megaloblastic changes, some of which presented with pseudopods, and parvovirus B19 was positive in a PCR analysis of her blood, which was compatible with parvovirus B19-induced hemophagocytic syndrome. Skin excisional biopsy of the nodule on the left forearm revealed a heavy inflammatory cell infiltrate throughout whole layers of a medium-sized vessel, the characteristic feature of PAN. PCR analysis of the vasculitis tissue showed a positive result for parvovirus B19.

**Interventions:**

Her symptoms spontaneously resolved with supportive care.

**Outcomes:**

She underwent regular follow-up without recurrence of vasculitis-associated symptoms.

**Lessons:**

This case highlights the presence of parvovirus B19 DNA in vasculitis tissues, which can support the role of cellular immune response in the pathogenesis of parvovirus-associated PAN.

## Abstract 2

Supplemental Digital Content is available in the text

## 1 Introduction

Polyarteritis nodosa (PAN) is necrotizing arteritis predominantly affecting medium-sized arteries without glomerulonephritis.^[1]^ Although the pathogenesis of PAN remains unclear, viral infections are known to be one of the major pathogenic triggers.^[2]^ Parvovirus B19 eliciting mainly febrile illness, polyarthritis or pure red cell aplasia has been reported to be one of the causes of PAN.^[3–6]^ However, there is no clear answer about whether the virus directly invades endothelial cells or if activated immune cells or the immune complex produced by parvovirus B19 is involved in the development of PAN.^[2]^ The presence of parvovirus B19 DNA in the vasculitis tissue can be a key clue to understand the pathogenesis of parvovirus-related PAN, but there has been conflicting reports about the identification of parvovirus DNA in tissue.^[7,8]^ Here, we describe a case of PAN in a patient with hemophagocytic syndrome associated with parvovirus B19 infection, where we confirmed the presence of parvovirus B19 DNA in the vasculitis tissue.

## 2 Case report

**Figure 1:**
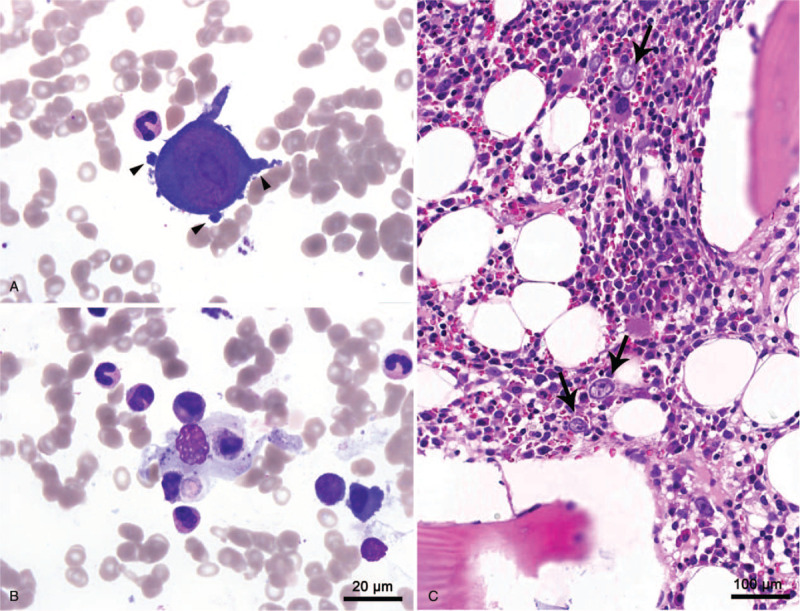
Bone marrow aspirate showed giant proerythroblasts with intranuclear inclusions and dog-ear-like cytoplasmic projections (arrowheads) (A) and features of hemophagocytosis, showing neutrophils engulfed by macrophages (Wright's stain, x1000) (B). Bone marrow biopsy was quite cellular, with a focal increase of giant proerythroblasts with prominent intranuclear eosinophilic inclusion bodies (arrow) (C).

**Figure 2:**
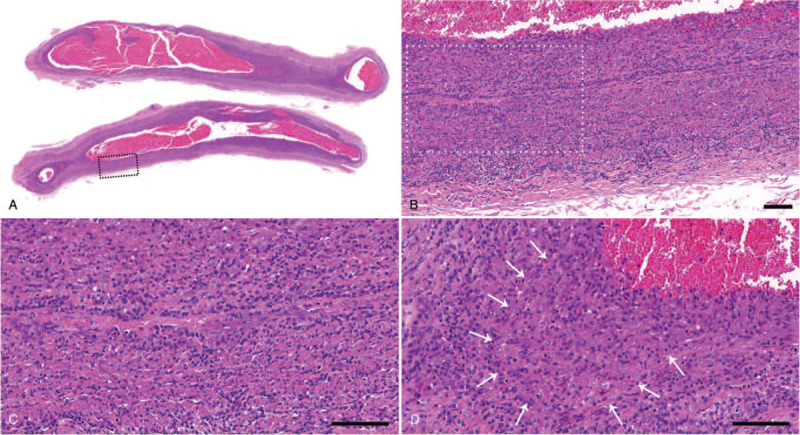
Excisional skin biopsy of the forearm shows (A, B, C) an intense infiltration of lymphoplasmacytic cells throughout whole layers of a medium-sized artery and (D) focal vague granulomatous reactions (arrow) characterized by an aggregation of epithelioid histiocytes. The area of the black rectangle in panel A is enlarged as panel B, and the white rectangle in panel B is shown in panel C. The scale bars represent 100 μm.

**Figure 3:**
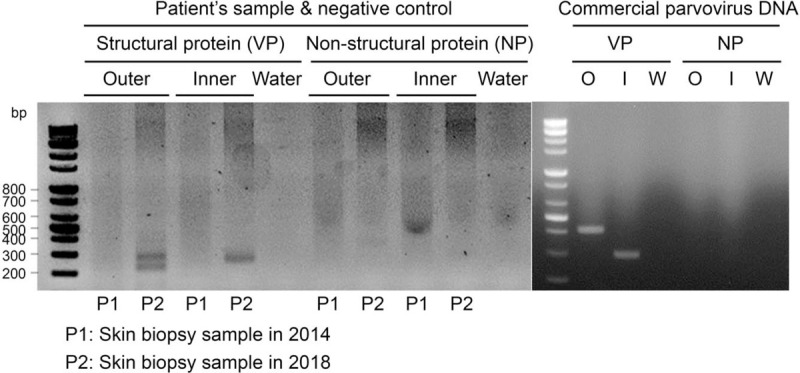
Parvovirus B19 DNA identification in the cutaneous vasculitis tissue. Agarose gel electrophoresis of PCR products from cutaneous vasculitis tissue obtained in 2018 (P2) shows a positive band only in the structural protein (VP) of parvovirus B19 at the size of approximately 290 bp (line 4 for inner primer). The double band by the outer primer for structural protein is judged to be a nonspecific band or primer-dimer (line 2). Commercial parvovirus DNA was used as a positive control. A skin biopsy sample from 2014 (P1) and DEPC-treated ultrapure water (Water) were used as negative controls.

A 38-year-old Korean woman presented to the Emergency Department with a 5-day history of fever. Initial laboratory tests revealed bicytopenia marked by a white blood cell count of 1,470/μL, hemoglobin of 7.6 g/dL, and platelets of 213 × 10^3^/μLL; serum ferritin and lactate dehydrogenase were elevated to 669 ng/mL (normal range, 10–120) and 443 U/L (208–378), respectively. A computed tomography (CT) scan of the abdomen showed moderate splenomegaly. Bone marrow aspirate revealed proerythroblasts with abnormal megaloblastic changes, some of which presented with pseudopods and pure red cell aplasia, and hemophagocytic features (Fig. 1). Based on the bone marrow aspirate study, a polymerase chain reaction (PCR) assay for parvovirus B19 virus DNA was conducted on her blood, which showed a positive result. At the time of the bone marrow aspiration, an abnormal liver function test was noted, with aspartate aminotransferase (AST) of 199 U/L (<37) and alanine aminotransferase (ALT) of 324 U/L (<41). Soluble IL-2 receptor (CD25) was elevated to 700 U/mL (158–623), but fibrinogen, triglyceride and NK cell activity were within normal ranges of 287.1 mg/dL (170–410), 47 mg/dL (<150), and 20.48%, respectively. She was diagnosed with secondary hemophagocytic lymphohistiocytosis and pure red cell aplasia associated with parvovirus B19 infection, and her symptoms spontaneously resolved without specific treatment. At the time of discharge, she complained of a painful palpable nodule in the left forearm, and an excisional biopsy was performed, which revealed vasculitis with intense lymphoplasmacytic infiltration throughout whole layers of a medium-sized artery, a feature of PAN (Fig. 2). To identify the presence of parvovirus DNA in vasculitis tissue, PCR products from vasculitis tissue were subjected to agarose gel electrophoresis. Amplification of parvovirus B19 DNA with inner primers (Supplementary Table) for structural sequence showed a positive band at a size of approximately 290 bp, which was also observed in the commercial positive control for parvovirus B19 (Amplirun, Vircell, Granada, Spain). However, the band of the outer primer, which amplified structural protein DNA at 410 bp, showed a double band at 290 and 250 bp, suggesting nonspecific bands or primer-dimers (Fig. 3).

## 3 Discussion

Parvovirus B19 primarily infects the epithelial cells of the respiratory tract through aerosol inhalation and disseminates to its main target of erythroid progenitor cells in bone marrow.^[9]^ In addition to bone marrow, the parvovirus B19 antigen is also detected in synovial tissues of chronic arthritis, in which it is mainly distributed in follicular dendritic cells, macrophages and lymphocytes, but not in synovial lining cells or endothelial cells.^[10]^ The absence of parvovirus B19 in endothelial cells and synovium, which is known to express the primary receptor Gb4Cer (globoside) for parvovirus B19, suggests that the virus is unlikely to invade directly into the synovial tissue.^[11]^ It is noteworthy that parvovirus B-19 isolated from synovial tissues is functionally active and can infect other macrophages or lymphocytes.^[10]^ This evidence suggests that monocytes infected by parvovirus B-19 in the bone marrow may spread over the target tissues, where B-19 infects other monocyte-lineage cells and elicits an inflammatory response.

There has been prior evidence of PAN as an immune complex-mediated disease based on the high prevalence of IgM antiphosphatidylserine–prothrombin complex and immunoglobulin M and C3 deposits in vasculitis tissues.^[14,15]^ Under this immune complex-mediated hypothesis, no virus will be detected in the vasculitis tissue. The loss-of-function mutation of adenosine deaminase type 2 (ADA2) is known to be associated with autosomal recessive PAN.^[12]^ A recent in-depth study revealed that the accumulation of adenosine by ADA2 mutation can enhance the formation of neutrophil extracellular traps (NET), which leads to the robust activation of macrophages and the subsequent production of TNFα in patients with ADA2 deficiency.^[13]^ In this paper, we have demonstrated the presence of parvovirus B19 DNA in vasculitis tissue, implying a role for the cellular immune response in the pathogenesis of PAN that involves more than immune complex-mediated inflammation. Although the exact mechanism is unknown, it is expected that the parvovirus B19 that is phagocytosed by immune cells in the bone marrow and moves to the vascular tissue will stimulate NET formation or TNFα production, contributing to the development of PAN.

## 4 Conclusion

This case provides direct evidence of the presence of parvovirus B19 DNA in vasculitis tissues, which can support the role of the cellular immune response in the pathogenesis of parvovirus-associated PAN more than immune complex-mediated inflammation.

## Author contributions

**Conceptualization:** Ji Yun Jeong, Ji Young Park, Ki Tae Kwon, Seungwoo Han.

**Data curation:** Ji Yun Jeong, Ji Yeon Ham, Seungwoo Han.

**Formal analysis:** Ji Yun Jeong, Ji Young Park, Ki Tae Kwon, Seungwoo Han.

**Investigation:** Ji Young Park, Seungwoo Han.

**Methodology:** Ji Yun Jeong, Ji Yeon Ham, Ki Tae Kwon, Seungwoo Han.

**Resources:** Ji Yun Jeong, Ji Young Park, Ji Yeon Ham, Ki Tae Kwon, Seungwoo Han.

**Validation:** Ji Yun Jeong, Seungwoo Han.

**Visualization:** Ji Yun Jeong, Ji Yeon Ham, Seungwoo Han.

**Writing – original draft:** Ji Yun Jeong, Ki Tae Kwon, Seungwoo Han.

**Writing – review & editing:** Ji Young Park, Ji Yeon Ham, Seungwoo Han.

## Supplementary Material

Supplemental Digital Content
