# Supplementary material for: Molecular evidence of parvovirus B19 in the cutaneous polyarteritis nodosa tissue from a patient with parvovirus-associated hemophagocytic syndrome: Case report
Source: Medicine (Baltimore). 2020 Sep 4;99(36):e22079. doi: 10.1097/MD.0000000000022079 (PMC7478508; doi:10.1097/MD.0000000000022079)
Supplement: Supplemental Digital Content [file medi-99-e22079-s001.docx]

**Molecular evidence of parvovirus B19 in the cutaneous polyarteritis nodosa tissue from a patient with parvovirus-associated hemophagocytic syndrome: Case report**

Ji Yun Jeong, MD., PhD.^1^, Ji Young Park, MD., PhD.^1^, Ji Yeon Ham, M.D., PhD.^2^, Ki Tae Kwon, MD., PhD.^3^, and Seungwoo Han, MD., PhD.^3^*

Supplementary table. PCR primers used for the amplification of parvovirus B19 in this study.

| Targets | Primer sequence (5' to 3') | | Length |
| --- | --- | --- | --- |
|  | Forward | Reverse |  |
| Structural sequence | | | |
| Outer | GGA CTG TAG CAG ATG AAG AG | TAT GGG ACT GAT GGT G | 410 |
| Inner | GGG TTT CAA GCA CAA GTA G | CCT TAT AAT GGT GCT CTG GG | 290 |
| Nonstructural sequence | | | |
| Outer | GGC AGC ATG TGT TAA GTG G | CAG TTG TTG TAG TGT TCC C | 369 |
| Inner | AAT ACA CTG TGG TTT TAT GGG CCG | CCA TTG CTG GTT ATA ACC ACA GGT | 284 |
